# Environmental contamination by vancomycin resistant enterococci (VRE) in Swedish broiler production

**DOI:** 10.1186/1751-0147-51-49

**Published:** 2009-12-02

**Authors:** Oskar Nilsson, Christina Greko, Björn Bengtsson

**Affiliations:** 1 National Veterinary Institute, Uppsala, Sweden; 2 Department of Clinical Sciences, Swedish University of Agricultural Sciences, Uppsala, Sweden

## Abstract

**Background:**

Vancomycin resistant enterococci are a frequent cause of nosocomial infections and their presence among farm animals is unwanted. Using media supplemented with vancomycin an increase in the proportion of samples from Swedish broilers positive for vancomycin resistant enterococci has been detected. The situation at farm level is largely unknown. The aims of this study were to obtain baseline knowledge about environmental contamination with vancomycin resistant enterococci in Swedish broiler production and the association between environmental contamination and colonisation of birds.

**Methods:**

Environmental samples were taken before, during and after a batch of broilers at three farms. Samples were cultured both qualitatively and semi-quantitatively for vancomycin resistant enterococci. In addition, caecal content from birds in the batch following at each farm was cultured qualitatively for vancomycin resistant enterococci.

**Results:**

The number of samples positive for vancomycin resistant enterococci varied among the farms. Also the amount of vancomycin resistant enterococci in the positive samples and the proportion of caecal samples containing vancomycin resistant enterococci varied among the farms. Still, the temporal changes in environmental contamination followed a similar pattern in all farms.

**Conclusion:**

Vancomycin resistant enterococci persist in the compartments even after cleaning and the temporal changes in environmental contamination were similar among farms. There were however differences among farms regarding both degree of contamination and proportion of birds colonized with vancomycin resistant enterococci. The proportion of colonized birds and the amount of vancomycin resistant enterococci in the compartments seems to be associated. If the factor(s) causing the differences among farms could be identified, it might be possible to reduce both the risk for colonisation by vancomycin resistant enterococci of the subsequent flock and the risk for spread of vancomycin resistant enterococci via the food chain to humans.

## Background

Vancomycin resistant enterococci (VRE) were first isolated in 1986 [1,2]. Since then, VRE have become endemic at many hospitals and are now considered a significant cause of nosocomial infections, mainly in immunocompromised patients [3]. In the early 1990s many farm animals in Europe were colonized with VRE. This was associated with extensive use of the glycopeptide avoparcin as a growth promoter [4], a use that was discontinued in the European Union in 1997 (Commission Directive 97/6 EC). In Sweden, avoparcin was only used for some years in the late 1970s and early 1980s [5,6] which could explain why VRE were not isolated from Swedish farm animals in the mid 1990s [7,8]. Later all use of growth promoters in Sweden was discontinued in 1986.

Vancomycin resistance is still rare among randomly selected enterococci isolated from farm animals in Sweden. However, using media supplemented with vancomycin an increase in the proportion of VRE-positive samples from Swedish broilers has been detected since 2000 [9]. It was shown that the increase is due to the spread of one clone of *vanA*-carrying *Enterococcus faecium *which has taken place in an apparently non-selective environment. In Swedish broiler production therapeutic use of antimicrobials is rare and instead the emphasis is on disease control by biosecurity. A farm to fork concept is applied to the control of food borne pathogens. Since VRE constitute a pool of resistance genes with possible implications for human healthcare, their occurrence in broiler production should if possible be contained. To this end, knowledge about colonisation of birds and environmental contamination at farm level is imperative.

Both VRE colonisation of broilers and contamination of farm environments has been studied elsewhere [10-12]. However, the almost monoclonal situation and low-level colonisation by VRE indicate a distinct epidemiological situation in Swedish broiler production. Therefore, the aims of this study were to obtain baseline knowledge about environmental contamination with VRE in Swedish broiler production and the association between environmental contamination and colonisation of birds.

## Methods

### Sampling

Three conveniently located broiler farms were chosen out of farms that previously had had broilers colonized with *van*A-carrying *E. faecium *(unpublished data). The three farms were chosen because they were similar in structure and size (i.e. number of houses and amount of broilers produced) and because the farmers were willing to participate. Each farm had four compartments and a total floor surface area between 5 200 and 7 000 m^2^. Within farms, hygiene barriers, including changing of shoes, were in place and each compartment had separate ventilation. During the study period, no flock was given any antibiotic treatment apart from the anticoccidial agent narasin which was used in feed until 5 days prior to slaughter.

#### Environmental samples

Environmental samples for culture of VRE were taken at 7 occasions (S1-S7) and on each sampling occasion, 2-5 samples from each compartment were taken (Table 1). All samples were collected from the end of March until the beginning of July 2007. At S1 and S7 the compartments had been cleaned and were ready for the subsequent batch of birds except that the bedding was not in place. The samplings S2-S4 took place approximately 1, 2 and 3 weeks after arrival of birds, S5 took place 2-4 days before slaughter and S6 after loading the birds for slaughter but before cleaning of the compartments. Birds were slaughtered when they were 36 to 43 days old. Exact day of sampling was chosen to minimize time of sample transport. Initial sampling (S1) at each farm was made by one of the researchers (ON) and thereafter by the farmers according to oral and written instructions. Briefly, floor samples were obtained with "Sterisocks humid" (SodiBox, Névez, France) by walking back and forth two times in the compartment, covering a distance of approximately 300 - 400 meters. The socks were made of jersey material that was factory pre-moistened with 15 mL distilled water. They were used outside sterile boot-covers and covered the entire sole of the boots. Other environmental samples were taken with sterile cloths (Sterile cloth, SodiBox), factory pre-impregnated with buffered peptone solution with 10% neutralising agent (lecithin, Tween 80, L-histidine, and sodium thiosulfate). Samples from air inlet and air outlet were obtained by wiping a surface area of approximately 0.04 and 0.2 m^2 ^respectively. Samples from the water- and feedline were obtained by wiping 5 meters of the line and the adjacent nipples. After sampling, each sock and cloth was placed in a separate plastic sampling bag and sent to the laboratory by mail, no later than the following day. Until mailing, samples were stored at 6°C.

**Table 1:** Results of bacteriological culture for vancomycin resistant enterococci of environmental samples.

				Farm A				Farm B				Farm C			
				
				1	2	3	4	1	2	3	4	1	2	3	4
		**Floor**		+ (1.7)	+ (1.8)^#^	+ (2.3)	+ (1.0)	-	-	+*	-	-	-	-	-
**Before arrival**		**Air inlet**		+ (1.4)	+ (1.3)^#^	+ (3.3)	+ (1.7)	-	+ (0.9)	+ (0.3)	+	-	+*	-	+
**of birds**		**Air outlet**		+ (1.5)	+ (0.8)	+ (1.4)	+ (2.1)	+ (1.3)*	+ (0.5)	+ (1.4)	+ (0.5)	-	-	-	-
**(S1)**		**Feed line**		+ (2.1)	+ (1.9)	+ (3.0)*	+ (3.5)	-	+ (1.3)	+ (0.0)	+ (0.7)*	+ (1.6)*	+ (0.6)	-	+ (0.5)*
		**Water line**		+ (1.3)*	+ (2.3)	+ (2.0)	+ (1.6)^#^	+ (1.1)	+*	+	-	-	-	-	-

**6-8 days after**		**Floor**		-	+	-	-	-	-	-	-	-	-	-	-
**arrival of birds**		**Air inlet**		+ (3.3)^#^	+ (3.2)*	+ (3.9)^#^	+ (3.1)	+ (1.7)	+ (1.4)	+*	+	-	+ (0.0)^#^	-	-
**(S2)**		**Air outlet**		+ (2.8)	+ (3.0)	+ (3.2)	+ (2.9)*	+ (1.3)*	+ (0.6)*	+ (1.9)	+ (0.6)*	+ (0.0)*	+^#^	-	-

**13-15 days after**		**Floor**		+ (3.3)*	+ (3.7)*	-	+ (4.2)	-	-	-	-	-	-	-	-
**arrival of birds**		**Air inlet**		+ (4.1)	-	-	+ (4.2)^#^	+ (2.6)^#^	+ (0.5)^#^	-	-	-	+ (0.6)*	-	-
**(S3)**		**Air outlet**		+ (4.1)	+ (4.2)	+ (4.2)*	+ (4.2)	+ (2.3)	-	+ (0.7)*	+ (0.6)*	+ (0.8)^#^	-	-	-

**20-22 days after**		**Floor**		+ (3.9)	+ (4.0)	+ (4.5)	+ (4.1)	-	-	+ (2.0)	+ (0.9)*	+ (2.2)*	+ (1.3)	-	-
**arrival of birds**		**Air inlet**		+ (4.1)^#^	+ (4.0)*	+ (4.3)	+ (4.2)	+ (2.4)*	+ (0.3)	+ (0.3)	+ (0.6)	+ (0.5)	+ (0.3)	-	-
**(S4)**		**Air outlet**		+ (3.8)	+ (4.1)	+ (4.1)^#^	+ (4.0)*	+ (2.6)	+ (2.9)*	+ (0.9)*	+ (1.5)	+ (0.8)	+ (0.8)*	-	-

**2-4 days**		**Floor**		+ (4.6)	+ (4.7)*	+ (4.9)	+ (4.6)^#^	+ (3.1)*	+ (3.0)*	+ (0.6)	+ (3.1)^#^	+ (2.6)	+ (2.1)^#^	-	+*
**before slaughter**		**Air inlet**		+ (4.5)	+ (4.6)	+ (4.6)^#^	+ (4.3)	-	-	+ (0.5)	-	+ (2.9)*	+ (0.0)	+ (0.6)^#^	+ (1.1)
**(S5)**		**Air outlet**		+ (4.6)*	+ (4.5)	+ (4.5)	+ (4.3)	-	+ (3.5)	+ (0.9)*	+ (3.4)	-	-	-	-

**After loading for**		**Air inlet**		+ (4.6)	+ (4.6)*	+ (4.6)	+ (4.4)	-	-	+ (0.9)	+ (0.8)^#^	+*	+ (2.5)	-	+ (0.0)*
**slaughter (S6)**		**Air outlet**		+ (4.5)*	+ (4.5)	+ (4.3)*	+ (4.4)*	+ (0.3)*	-	+ (0.9)*	+ (1.9)	+ (0.0)	+ (3.5)*	-	-

		**Floor**		+ (3.6)	+ (2.8)^#^	+ (3.7)	+ (3.5)	-	-	+^#^	-	+ (0.5)	+ (0.9)	+ (1.1)*	+ (1.1)
**Before arrival**		**Air inlet**		+ (3.5)*	+ (3.2)^#^	+ (3.8)	+ (2.9)	ns	+ (1.3)^#^	-	+*	-	-	+ (0.5)	-
**of new birds**		**Air outlet**		+ (3.6)	+ (3.1)	+ (3.9)	+ (3.9)	ns	+ (0.0)	+	+ (0.0)	-	+ (1.6)	-	+ (0.3)*
**(S7)**		**Feed line**		+ (3.1)	+ (2.9)	+ (2.9)*	+ (2.8)^#^	ns	+ (1.5)	-	+ (2.1)	+ (1.7)*	+ (1.2)	-	-
		**Water line**		+ (2.6)	+ (2.3)	+ (3.1)	+ (1.9)	ns	+ (1.2)	-	-	+	+ (1.2)*	-	-

+ = positive sample, - = negative sample, ns = sample not taken. Numbers in brackets indicate the amount of VRE (log number of colony forming units/plate, adjusted for dilution) in samples positive on direct plating.

* = isolates identified to species, ^# ^= isolates identified to species and analysed with MLST.

#### Caecal samples

From the batches of broilers following the environmental sampling period, 10 caecas per group of birds slaughtered (slaughter group) were sampled. Caecas were collected at the slaughterhouse before the birds were scalded and sent to the laboratory by mail on the same day.

### Bacterial isolation, identification and counting

#### Environmental samples

Samples arrived at the laboratory the day after mailing and were analysed on the day of arrival or at the latest the following day. Samples were cultured both for qualitative and semi-quantitative detection of VRE. First, Enterococcosel (Merck, Darmstadt, Germany) was added to the samples (25 mL to cloths and 50 mL to socks) which were then placed in a Stomacher (Stomacher^®^-80 Biomaster lab system, Seward Ltd., Worthing, United Kingdom) and treated for 1 minute. Thereafter, 10 mL of the solution was removed and divided in two aliquots. For semi-quantitative detection (degree of contamination), 0.1 mL from one aliquot was streaked on Slanetz-Bartley agar (Oxoid, Basingstoke, UK) supplemented with vancomycin (16 mg/L) (Sigma-Aldrich, Steinheim, Germany). For qualitative detection (presence of VRE), the other aliquot was pre-enriched at 37°C for 3-4 hours with the primary aim of resuscitating injured bacteria. Next, 0.1 mL was streaked on Slanetz-Bartley agar (Oxoid) supplemented with vancomycin (16 mg/L) (Sigma-Aldrich). The plates were then incubated at 37°C for 48 hours. The number of colonies with morphology consistent with enterococci from the non pre-enriched aliquot was recorded. If the number of colonies was too high for accurate counting the aliquot was diluted 1:10 and 1:100 and re-cultured as above. From the pre-enriched aliquot only growth or non-growth of colonies with morphology consistent with enterococci was recorded. From all positive samples at least one colony was sub-cultured on blood agar (Oxoid) and Bile-Esculine agar (Oxoid) and incubated at 37°C for 24 hours. Colonies with morphological appearance typical for enterococci on all media and positive reaction on Bile-Esculine agar were considered as *Enterococcus *sp. Isolates were stored at -70°C for further investigations.

#### Caecal samples

Caecal samples were cultured as previously described [9]. Briefly, caecal content (0.5 grams) was suspended in 4.5 mL saline from which 0.1 mL was streaked on Slanetz-Bartley agar (Oxoid) supplemented with vancomycin (16 mg/L) (Sigma-Aldrich) and incubated at 37°C for 48 hours. Samples with growth of colonies with morphology consistent with enterococci were handled as above.

### Species identification

Species identification was done according to Devriese et al [13]. Environmental isolates chosen for multilocus sequence typing (MLST) analysis (see below) were included along with additional isolates so that at least one isolate, if existing, from each compartment and sampling occasion was included (n = 77). In addition, two caecal isolates per slaughter group were included (n = 8). Both additional environmental isolates and caecal isolates were selected at random within compartments and slaughter groups. The reference strain *Enterococcus faecalis *ATCC 29212 was used for quality control.

### Susceptibility testing

All stored environmental and caecal isolates (n = 214) were tested for susceptibility to vancomycin by determination of MIC using micro dilution in broth according to the standards of the Clinical and Laboratory Standards Institute [14]. Tests were performed in cation adjusted Mueller-Hinton broth (Difco, Sparks, USA) using VetMIC™ panels (SVA, Uppsala, Sweden). The reference strain *Enterococcus faecalis *ATCC 29212 was used for quality control.

### Multilocus sequence typing (MLST)

Among the stored environmental isolates (n = 189) 24 were selected at random and analysed with MLST as described by Homan et al [15], with modifications according to the MLST web site [16].

### Statistical analysis

Absolute numbers of colonies from semi-quantitative detection (degree of contamination) in environmental samples were transformed to logarithmic values before statistical analysis. All analyses for environmental and caecal samples were done by Pearson's χ^2 ^test using Stata software (release 10, Stata, College Station, TX, USA). Statistical significance was set as p = 0.05.

## Results

### Sampling, bacterial isolation and counting

#### Environmental samples

The number of VRE-positive samples differed among the farms (Table 1). For each farm, the proportions of VRE-positive samples in total and on direct plating were: Farm A 94% and 93%; Farm B 64% and 54%; and Farm C 42% and 34%. Also the degree of contamination measured by semi-quantitative detection differed among the farms (Table 1).

At the first sampling (S1) VRE were present in the environment at all farms, but the number of positive samples and the degree of contamination varied among farms. At Farm A, VRE were detected on direct plating in all 20 samples taken initially; whereas at Farm C, VRE were only detected in 5 of the samples, of which only 3 were positive on direct plating (Table 1).

The amount of time before VRE were detected in the floor samples taken during the batch (S2-S5) varied both among farms and among compartments at the same farm. At Farm A, VRE were detected in floor samples from 1 of 4 compartments 1 week after arrival of birds, and in 3 of 4 compartments 2 weeks after arrival of birds. In contrast, at Farm B and Farm C, VRE were not detected in floor samples until 3 weeks after arrival of birds. Even though VRE were detected in floor samples from all but 1 compartment 2-4 days before slaughter, the degree of contamination varied between farms (Table 1).

At the first and the last sampling (S1 and S7) the number of positive samples was equal in 7 of the 11 compartments where sampling was completed according to schedule. However, in all of these 7 compartments more samples were positive on direct plating or the degree of contamination measured by semi-quantitative detection was higher, after the batch compared to before. Of the remaining compartments, 3 (all on Farm C) had more VRE-positive samples and 1 (on Farm B) had fewer VRE-positive samples after the batch compared to before.

Among samples taken from cleaned compartments (S1 and S7), the feed line was the only sample, that with statistical significance predicted whether VRE could be detected in any sample from the compartment at that sampling occasion (χ^2 ^test, p = 0.05).

#### Caecal samples

At all three farms, birds from compartments 1 and 2 were slaughtered in one slaughter group and compartments 3 and 4 in another. The numbers of VRE-positive caecal samples were: from Farm A, 6 and 8 samples (70%); and from Farm B, 4 and 7 samples (55%). From Farm C VRE could not be isolated from any of the 20 caecal samples analysed. The differences between Farm C versus Farm A or Farm B was statistically significant (χ^2 ^test, p < 0.001).

### Species identification, susceptibility testing and MLST

All identified isolates (n = 85) were *E. faecium*, all susceptibility tested isolates (n = 214) had MIC for vancomycin of ≥128 mg/L, and all isolates (n = 24) investigated with MLST were of ST310.

## Discussion

The result of the species identification, susceptibility testing and MLST indicate that the VRE isolated from the study farms belong to the *vanA*-carrying *E. faecium *clone previously described to dominate among Swedish broilers [9].

Even though VRE were isolated in all compartments at all farms we found that environmental contamination with VRE at the three farms differed. Not only did the proportion of VRE-positive samples vary among the farms but also the degree of contamination. Differences among the farms were also seen in samples from individual chickens. VRE could not be detected in caecal samples from the farm with the lowest proportion of VRE-positive samples and the lowest degree of environmental contamination (Farm C) whereas from the other two farms 70% and 55% of the caecal samples were VRE-positive. This indicates an association between the degree of environmental contamination and colonisation of birds.

Although the degree of environmental contamination varied, the temporal changes in contamination followed a similar pattern in all farms. At the start of the study, when cleaned and empty compartments were sampled (S1), VRE were present in all but one compartment. That VRE persist even after cleaning and disinfection is in agreement with previous studies [10-12]. At all farms the degree of contamination increased during the batch and then decreased when the compartments were again cleaned after the batch. However, in floor samples taken when birds were present in the compartments (S2-S5), bedding and faeces stuck to the socks and were included in the samples. In such cases, the sample volume was larger than from empty floors, which could partly explain the apparent reduction of VRE in floor samples from S5 to S7. For samples from Air inlet and Air outlet the difference in the amount of material was negligible. Still, VRE were not eliminated from any of the compartments. In addition, two of the farms had a higher degree of VRE contamination after the studied batch, indicating that the cleaning routines are not sufficient, which could lead to a build-up of VRE within the compartments. However, it cannot be excluded that the higher degree of VRE contamination after, as compared to before the batch (S7 to S1) was influenced by climate factors. In empty compartments the ventilation is turned down and temperature and humidity could be affected by the outside climate. The study period was in the spring to early summer and the temperature in the empty compartments was probably lower at S1 than at S7 which could influence the degree of VRE contamination detected.

It has been suggested that VRE persisting in the compartments subsequently colonize the following batch of broilers [11]. Our study indicates that even the low degree of VRE contamination seen on Farm C at the start of the study (S1), is sufficient for amplification and spread. As soon as birds are put in to the compartments they would start to become colonized with the persisting VRE. Borgen et al [11] isolated VRE from faecal samples in 3 of 5 study units already after 1 week and after 3 weeks all study units were VRE-positive. In our study, only 1 of 12 compartments had a VRE-positive floor sample one week after arrival of birds (S2). On the other hand, at that time the bedding mainly comprises of shavings and therefore only a small proportion of the floor samples were actually faeces which would have decreased the sensitivity. Nevertheless, in both studies the time before VRE colonisation could be detected varied among study units.

As time proceeds, more and more birds would become colonized with VRE leading to increased contamination, both in the bedding and in the rest of the environment. Accordingly, there was an increase in the degree of VRE contamination on the floors during the first weeks of the rearing period. Garcia-Migura et al [10] describes a similar increase until the broilers were three weeks old, but the percentage of VRE-positive faecal samples decreased in the end of the rearing period. Also studies by Devriese et al and Kaukas et al [17,18] indicate a decreased proportion of *E. faecium *in the intestinal flora of chickens with increasing age. As mentioned, the floor samples in our study should be regarded as environmental samples from the floors rather than actual faecal samples. Therefore the degree of colonisation of the birds in our study could have decreased without being reflected in the contamination of the floors. Still, even if the amount of VRE in the intestines of the birds is diminishing the VRE in the environment constitute a risk for later contamination of the carcasses. The skin and feathers of the birds will likely be contaminated by VRE from the environment, as indicated by a study finding elevated rates of enterococci in air samples taken behind running vehicles transporting poultry [19]. Furthermore, Rule et al [20] found enterococci in water samples from various places within poultry slaughter houses (e.g. scald tank and plucking facilities) implying that VRE on skin and feathers of the birds could contaminate the whole carcass is not unlikely.

## Conclusion

In conclusion, the main findings of this study are that VRE persist in the compartments even after cleaning and that the temporal changes in environmental contamination is similar among studied units. There were however differences among the farms regarding both degree of contamination and proportion of birds colonized with VRE. Furthermore, the proportion of colonized birds and the amount of vancomycin resistant enterococci in the compartments seems to be associated. If the factor(s) causing the differences in degree of contamination and proportion of birds colonized with VRE among farms could be identified, it might be possible to reduce the amount of VRE both at the farms and in the birds. Thereby, both the risk for VRE-colonization of the subsequent flock and the risk for spread of VRE to humans via the food chain by contaminated broiler carcasses would be reduced.

## Competing interests

The authors declare that they have no competing interests.

## Authors' contributions

The study was designed by all authors. ON did the field work and the laboratory work. ON drafted the manuscript and all authors revised, read and approved the final manuscript.
